# An Association between Rainy Days with Clinical Dengue Fever in Dhaka, Bangladesh: Findings from a Hospital Based Study

**DOI:** 10.3390/ijerph17249506

**Published:** 2020-12-18

**Authors:** Kazi Mizanur Rahman, Yushuf Sharker, Reza Ali Rumi, Mahboob-Ul Islam Khan, Mohammad Sohel Shomik, Muhammad Waliur Rahman, Sk Masum Billah, Mahmudur Rahman, Peter Kim Streatfield, David Harley, Stephen P. Luby

**Affiliations:** 1 International Centre for Diarrhoeal Disease Research, Bangladesh (icddr,b), Dhaka 1212, Bangladesh; rumi@icddrb.org (R.A.R.); khanmahboob@yahoo.com (M.-U.I.K.); mshomik@icddrb.org (M.S.S.); mwrahman@gmail.com (M.W.R.); billah@icddrb.org (S.M.B.); pkstreatfield@icddrb.org (P.K.S.); 2 North Coast Public Health Unit, New South Wales Health, Lismore, NSW 2480, Australia; 3 University Centre for Rural Health, University of Sydney, Lismore, NSW 2480, Australia; 4 Department of Biostatistics, Yale School of Public Health, New Haven, CT 06520-0834, USA; mayushuf@gmail.com; 5 Institute of Epidemiology, Disease Control and Research (IEDCR), Dhaka 1212, Bangladesh; mahmudur57@gmail.com; 6 Children’s Health Queensland Clinical Unit, Faculty of Medicine, The University of Queensland, Queensland Children’s Hospital, South Brisbane, QLD 4101, Australia; d.harley@uq.edu.au; 7 Center for Innovation in Global Health, Stanford University, Stanford, CA 94305, USA; sluby@stanford.edu

**Keywords:** dengue, seasonal variation, year-long transmission, climatic variability, rainfall, prediction, disease control campaigns

## Abstract

Background: Dengue, a febrile illness, is caused by a Flavivirus transmitted by *Aedes aegypti* and *Aedes albopictus* mosquitoes. Climate influences the ecology of the vectors. We aimed to identify the influence of climatic variability on the occurrence of clinical dengue requiring hospitalization in Zone-5, a high incidence area of Dhaka City Corporation (DCC), Bangladesh. Methods and Findings: We retrospectively identified clinical dengue cases hospitalized from Zone-5 of DCC between 2005 and 2009. We extracted records of the four major catchment hospitals of the study area. The Bangladesh Meteorological Department (BMD) provided data on temperature, rainfall, and humidity of DCC for the study period. We used autoregressive integrated moving average (ARIMA) models for the number of monthly dengue hospitalizations. We also modeled all the climatic variables using Poisson regression. During our study period, dengue occurred throughout the year in Zone-5 of DCC. The median number of hospitalized dengue cases was 9 per month. Dengue incidence increased sharply from June, and reached its peak in August. One additional rainy day per month increased dengue cases in the succeeding month by 6% (RR = 1.06, 95% CI: 1.04–1.09). Conclusions: Dengue is transmitted throughout the year in Zone-5 of DCC, with seasonal variation in incidence. The number of rainy days per month is significantly associated with dengue incidence in the subsequent month. Our study suggests the initiation of campaigns in DCC for controlling dengue and other *Aedes* mosquito borne diseases, including Chikunguniya from the month of May each year. BMD rainfall data may be used to determine campaign timing.

## 1. Introduction

Dengue is a febrile illness caused by dengue virus, a Flavivirus. The virus is transmitted from person to person by the mosquitoes *Aedes aegypti* and *Aedes albopictus* [1]. There are four different serotypes of dengue virus: DEN 1, 2, 3, and 4. Infection confers lifelong immunity to the infecting serotype. Subsequent infection with a different serotype may result in dengue haemorrhagic fever (DHF) or dengue shock syndrome (DSS) [2,3,4,5,6]. The classical form of dengue is characterized by high fever with bone pains. Plasma leakage, with or without haemorrhage, is the primary pathogenic mechanism for severe dengue. In severe dengue, a patient’s condition becomes critical on days 3–7 of illness, when the associated high fever subsides [2].

Global estimates of people at risk of dengue are between 2.5 and 3.6 billion, and annual cases of dengue are 50–390 million [3,7,8]. Around half a million dengue patients are hospitalized annually and case fatality is 2.5%. Three-quarters of the dengue cases come from the World Health Organization (WHO) South-East Asia (SEA) and Western Pacific regions [9]. In 2000, 7 of the 11 SEA region countries reported 63,000 dengue cases in total. This number almost tripled in 2010, with a total of 187,000 reported dengue cases from 10 of the 11 SEA region countries [9].

Dengue has been reported to be occurring in Dhaka, the capital of Bangladesh, since the 1960s [10,11,12]. Hospital based studies in Chittagong city during 1996–1997, and Rajshahi, Khulna, Sylhet, and Chittagong cities during 1999, concluded that 13–20% of febrile presentations were due to dengue [13,14]. A dengue outbreak in the year 2000 involved more than 5000 cases between July and December from Dhaka, Khulna, and Chittagong, with a case fatality rate of 1.7% [15]. During that outbreak, In Dhaka, the highest number of cases was reported from the suburbs of Zone-5 of Dhaka City Corporation (DCC). This outbreak followed the introduction of a new serotype (DEN 3) of dengue virus from neighboring Myanmar [16,17].

Several studies have modeled dengue incidence over time in Dhaka, along with forecasting [18] and finding space-time clustering of dengue [19], testing its association with river level [20], environmental temperature with its daily fluctuation [21], temperature and rainfall [22] and the climatic factors combined [23]. In our study, we explored the individual roles of the key ambient weather conditions—temperature, rainfall, and humidity—on the occurrence of dengue in Zone-5, a high incidence area of DCC.

## 2. Methods

### 2.1. Data Collection

We included hospitalized dengue cases from Zone-5 of DCC between 2005 and 2009 in our analyses. A person diagnosed with dengue on discharge from the hospital was designated a study case. This diagnosis was made by the hospital clinicians. A trained data extraction team consisting of a study medical officer, a research officer, and research assistants, extracted dengue case records from four major catchment hospitals of Zone-5 of DCC, including Central Hospital, Bangladesh Medical College Hospital, Dhaka Medical College Hospital and Holy Family Red Crescent Medical College Hospital. The data extraction team searched all the patient files from the study time period, identified the cases, and recorded information on them using a pro-forma. The hospital record extraction was carried out in 2009 and 2010.

We obtained average daily weather data including temperature, rainfall and humidity recorded in the weather station of the Bangladesh Meteorological Department (BMD) located in Dhaka.

### 2.2. Data Management and Analysis

We calculated monthly dengue hospitalizations. The number of days per month with greater than 3 mm of rain, the 25th percentile of daily rainfall in a month, were counted. We derived monthly average of the daily mean temperature and percent relative humidity. We imputed missing values for monthly average humidity in 2009, using the seasonal autoregressive integrated moving average (ARIMA) (1,0,0)(1,0,0)_12_ model as this provides minimum error among all univariate models.

We used median polish to see the changes in number of dengue hospitalizations over months and years [24]. This is a robust method that additively fits data in a two-way layout. Median polish estimates the row effects and column effects on the overall median through an iterative process. We used the sequence of months in a year as rows, and different years in columns. The effects of years were translated to the trend, and the effects of months were translated to the seasonality of dengue hospitalizations.

We used ARIMA models for the number of monthly dengue hospitalizations. We strictly followed the Box–Jenkins modeling strategy to select the parsimonious ARIMA models. First, we stabilized the mean and variance using suitable square root transformations for all the variables. We then identified the orders for the ARIMA, using the autocorrelation function (ACF) and partial autocorrelation function of the variables. For model selection, we also considered the minimum on the root mean squared forecast error (RMSFE) of the observed data and the Akike information criterion (AIC) [25].

We modeled climatic variables using ARIMA models. After that we removed the self-explanatory part from the time series data by deducting the modeled variable from the observed variable, which was defined as “whitened variable”. We used the cross correlation function of the pre-whitened climate variables and the dengue hospitalizations to identify the lags of climate variables contributing to the variability of the dengue outcomes. We regressed the total dengue cases on the climatic parameters that were identified to be causally linked with the lagged climatic variable. We used Poisson regression to estimate the model parameters, assuming that the number of dengue cases follows Poisson distribution.

### 2.3. Ethics

The study was approved by the Research Review Committee and Ethical Review Committee of International Centre for Diarrhoeal Disease Research, Bangladesh (icddr,b). Written permission was obtained from the hospital authorities before doing the hospital record extractions. We purchased weather data from BMD.

## 3. Results

**Figure 1:**
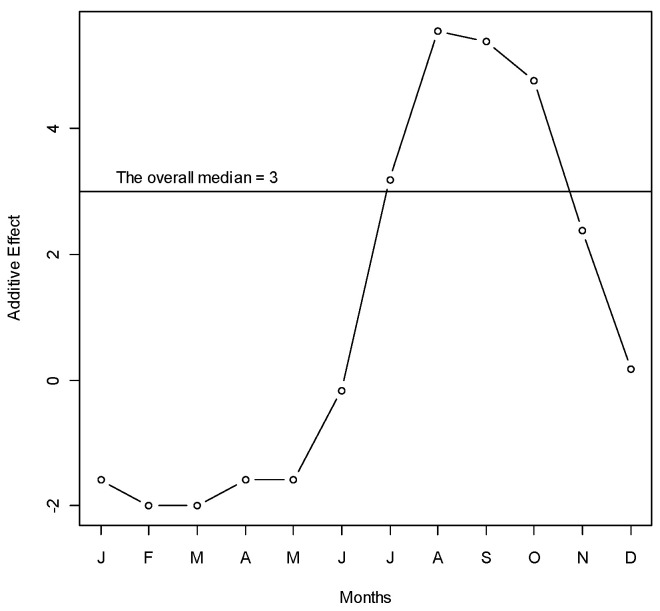
Median polish of the number of dengue cases over months during years 2005–2009 in Zone-5 of Dhaka City Corporation, Dhaka, Bangladesh. Y-axis represents the monthly additive effect of hospitalized dengue cases to the overall median in square root scale. X-axis represents the months. To get the median dengue cases in a specific month, add the overall median to the month’s effect and take the square. For example, the median number of dengue cases in June is (3 − 0)^2^ = 9 cases (approximately).

**Figure 2:**
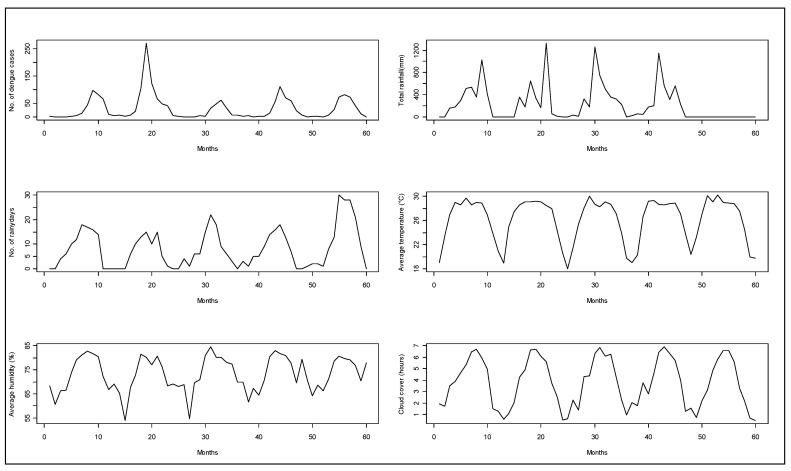
Monthly aggregated observations for hospitalized dengue cases from Zone-5 and weather parameters from 2005 to 2009 in Dhaka city.

A total of 1902 hospitalized patients with diagnosis of dengue from the Zone-5 catchment hospitals of DCC were included in the analysis. In those hospitals, there were a total of 324 cases in 2005, 701 cases in 2006, 198 cases in 2007, 355 cases in 2008, and 324 cases in 2009. We observed a seasonal pattern in dengue cases. The number of dengue cases rose sharply from June. It reached its peak in August and continued through September. After that, the frequency of dengue hospitalization declined. The dengue hospitalization occurred in the study area all through the months of the study years and we did not find any month where the hospitalization reached to zero (on average) (Figure 1). Median polish did not identify any trend of dengue incidence over the years, which was also reflected in the time series plot of dengue cases (Figure 2).

**Figure 3:**
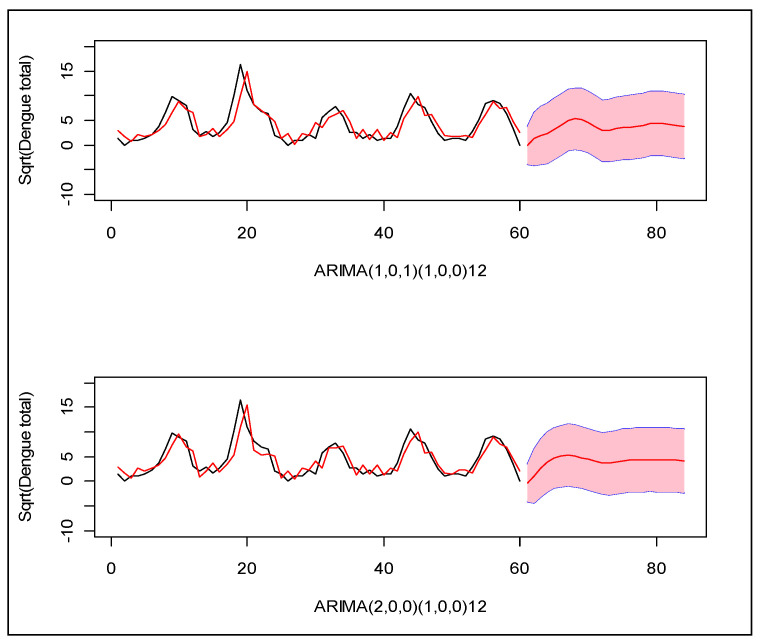
Two seasonal ARIMA models for the monthly number of dengue cases during years 2005–2009 in Zone-5 of Dhaka City Corporation, Dhaka, Bangladesh. X-axis indicates the index of the months, and Y-axis indicates the total number of dengue cases in square root scale. The black line is the observed cases and the red line is the modeled cases. The red line with pink region indicates the forecast of the next 20 months with 95% confidence band for prediction.

Following the Box–Jenkins model selection criteria [25], we identified two suitable models which can explain the monthly incidence of dengue in this region: (1) Seasonal ARIMA(1,0,1)(1,0,0)_12_ and (2) Seasonal ARIMA(2,0,0)(1,0,0)_12_ for square root transformed total dengue cases. Between the two, the second model provides the minimum error variance, root mean squared forecast error (RMSFE), and minimum Akike information criterion (AIC). Neither model closely predicted the observed patterns in prior years (Figure 3).

The dengue cases in a particular month increased by 13% of the total incident cases of the same month of the previous year (Seasonal AR_1_ = 0.13). Overall the average square root incidence of dengue was 4/month implying 16 cases/month in the original scale (Table 1). We used seasonal models such as ARIMA(1,0,0)(1,0,0)_12_ for average temperature, ARIMA(0,0,0)(1,0,0)_12_ for humidity, ARIMA (1,0,0,)(1,0,0)_12_ for rainy days. While looking at the cross correlation function (see Supplementary Materials) of the total dengue cases, with all other climatic variables, we only observe a meaningful correlation with the 1st lag of total rainy days per month and total dengue.

**Figure 4:**
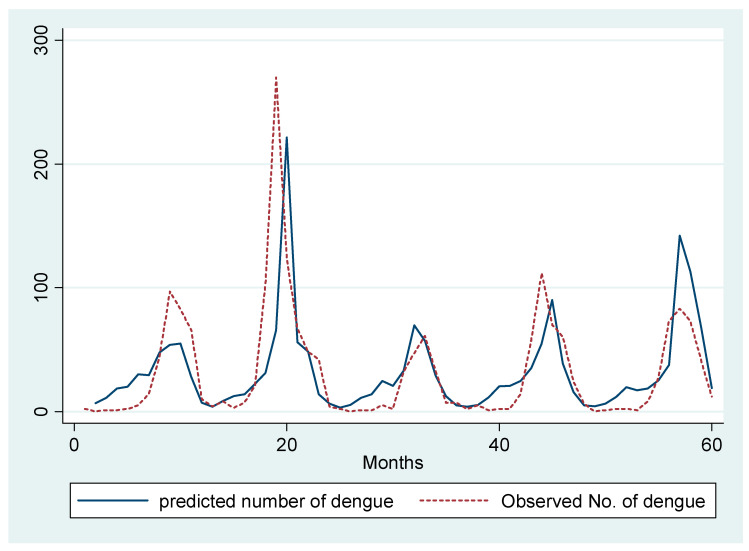
Prediction of total number of monthly dengue hospitalizations by Poisson regression model.

We ran a Poisson regression model of total dengue with the covariates including one-month lag of dengue, seasonal lag of dengue, and the total rainy days in the previous month. To control for confounding due to temperature, we have included the average temperature in our model as covariate. We observe that a one day increase in the total rainy days in a particular month increases the total monthly dengue cases by 6% in the succeeding month (RR = 1.06, 95% CI: 1.04–1.09). We also observe an around 1% increase in dengue incidence due to a one unit increase in the previous month’s total dengue cases (RR = 1.006, 95% CI: 1.003, 1.01) (Figure 4).

## 4. Discussion

Dengue diagnoses were made in the study hospitals all through the year, with a sharp increase in incidence from June, reaching its peak in August. Dengue incidence was significantly associated with the number of rainy days in the preceding month. An additional rainy day was associated with 6% increase in incidence. Our data also showed a sharp rise in annual dengue incidence in 2006, as compared to the other study years.

Seasonal variation in the incidence of dengue has been observed in other studies from Bangladesh. All studies find the highest incidence between July and October [18,19,26]. The number of febrile patients tested dengue positive reached a peak in the third quarter of the year [11,27]. The year-long transmission of dengue in Dhaka city, observed in our study, is consistent with a hospital based surveillance showing presence of dengue throughout the year [26]. *Aedes aegypti* breeds in artificial and natural water containers around dwellings, so during periods of low rainfall storage of water by householders might allow ongoing egg laying and transmission of dengue [28,29]. This finding warrants year-long surveillance for dengue. This also emphasizes the need for the physicians to be vigilant for dengue throughout the year.

In Dhaka, the monsoon rain starts from the month of June, and continues through September [30]. This corresponds with the continued upward trend of dengue incidence subsequently. The association of rainfall and dengue has also been observed in other studies from Bangladesh and the region at different lag periods. In Thailand, significant positive association was found between two months’ cumulated rainfall and dengue virus infection at a temperature more than 23.2 °C [31]. In Singapore, cumulative rainfall has been found to increase dengue incidence linearly, at a lag ranging from 5–20 weeks [32]. A recent analysis from Dhaka using data from a private diagnostic facility found a positive correlation between rainfall and the number of dengue cases at two-month lag period [22].

In our study, we used hospitalized dengue cases within a high incidence area of DCC. The shorter lag-period (one month) for rainfall influencing dengue incidence observed in our study can thus be more applicable to high dengue incidence areas in urban settings. Dengue has an extrinsic (within the mosquitoes) incubation period ranging from 5 to 33 days and an intrinsic (within human host) incubation period of 2 to 15 days [33]. A dengue patient starts having increased vascular permeability, resulting in severe dengue, in between days 4 to 7 after symptom onset [34]. Patients are likely to be hospitalized at this stage. The total of the extrinsic incubation period, the intrinsic incubation period, and the time from symptom onset of dengue to hospitalization, is approximately one month so the associations we found at one-month-lag are biologically and medically plausible.

We used number of rainy days instead of cumulative monthly rainfall in our study. Heavy rain may wash larvae out of breeding sites and consequently reduce incidence. As compared to the cumulative rainfall, the use of number of rainy days in a particular month better represents spread of rain over a month, resulting in favorable breeding places for the *Aedes* mosquitos.

Unlike our study, other studies have demonstrated an association between dengue incidence and temperature [31,32]. In two laboratory experiments in Thailand, increase in temperature from 30 °C to 32–35 °C reduced the extrinsic incubation period by five days [35]. Mosquito biting rate also varies with temperature. Although analysis showed the interaction between mean temperature and daily fluctuations of temperature influences dengue transmission at a lag of one month [21]; the small annual variation in temperature in Dhaka city may explain our inability to detect any association. We infer that rainfall rather than temperature variation determines seasonal variation in dengue incidence in Dhaka.

In our study, we actively extracted hospital records instead of analyzing passively reported hospital cases. We covered all the major catchment hospitals of Zone-5 of Dhaka that previously reported high incidence of dengue. Our study thus provides more complete and representative evidence of hospitalizations from dengue clinically diagnosed by the respective hospital physicians. The passive surveillance system that was in place for dengue fever during our study period was incomplete, as dengue reporting was requested only during the known dengue season coinciding with the monsoon [12]. Moreover, there was no validation of dengue reporting through that passive surveillance system, which still remains a problem resulting in under-estimation [36]. We thus decided to limit our analysis to our study data and period. Moreover, as we were investigating associations between climatic variability and dengue, we assumed that our data from the past would still provide robust evidence.

Relying on clinical diagnosis of dengue could result in misclassifications. Especially, having prior knowledge of the seasonal variation of the illness could result in more clinical diagnosis of the disease during the known dengue season in the country. Moreover, presence of other arboviral diseases like Chikungunya, presenting with similar symptoms in Dhaka city [37], would make diagnosis of dengue on the basis of clinical presentations more difficult. Studies involving laboratory confirmed dengue cases would thus provide more robust evidence for any association between climatic variability and dengue occurrence. In addition, future models should also take disease control measures into account. Our study could not control for them due to the unavailability of reliable data.

The role of rainfall, including number of rainy days, on the increasing occurrence of dengue has significant public health implications, particularly for Bangladesh. Based on our data, control of *Aedes* mosquito breeding and the consequent interruption of the transmission cycle should be enhanced in May. However, the timing of the activation of the increased control measures [38] should be guided by the rainfall pattern and the start of monsoon in a particular year. For example, in 2015, there was an early peak of rainfall in April [39]. The dengue control activities should have been accelerated from that month for that particular year. This is why inter-agency collaboration, including Bangladesh Meteorological Department, is crucial to implement the action measures in response to changes in rainfall [40]. Preparatory measures could even be taken based on future prediction of rain. The control measures including strong public messaging should involve home-based (removing indoor potted plants, draining of refrigerator trays, regular complete emptying of plastic drums, increased bed net use), peri-domestic (draining of water from or removal of tires, vehicle parts, and discarded construction materials) and outdoor (vector control measures in public parks) interventions [41,42]. All these will also help prevent the occurrence of other *Aedes* mosquito borne diseases like Chikungunya, which caused large outbreaks in both rural and urban Bangladesh in recent years [37,43].

## 5. Conclusions

Our study has demonstrated significant association between rainfall (especially number of rainy days) and dengue occurrence in Dhaka city, at one-month lag in a high incidence area. A year-long transmission of dengue has also been observed. The communicable disease control program of the country should take this evidence into account for better prevention and control of dengue, as well as other *Aedes* mosquito borne diseases.

## Figures and Tables

**Table 1:** ARIMA models and parameters estimates for each of the variables under study.

Variables *	Models	Intercept	AR1 **	AR2	MA1	SAR1	RMSFE	AIC
Total Dengue	ARIMA (1,0,1)(1,0,0)_12_	4.10	0.64	-	0.35	0.27	1.96	263
	ARIMA (2,0,0)(1,0,0)_12_	4.30	1.15	−0.49	-	0.13	1.86	257
Temperature	ARIMA (1,0,0)(1,0,0)_12_	5.10	0.65	-	-	0.84	0.13	−51.3
Rainy days/month	ARIMA (1,0, 0,)(1,0,0)_12_	2.42	0.58	-	−0.13	0.66	0.92	178.6
Humidity		4.29	-	-	-	0.8	0.06	−153

* The variable were reported in square root transformed scale. ** AR1—autoregression at 1 month lag; MA—moving average; SAR—seasonal autoregression.

## Supplementary Materials

The following are available online at https://www.mdpi.com/1660-4601/17/24/9506/s1, Figure S1: Cross Correlation Function (CCF) plot for different climate variables and the total number of monthly dengue cases for years 2005-2009 in Dhaka City Corporation, Dhaka, Bangladesh. Figure S2: Autocorrelation and partial autocorrelation function plots for total number of monthly dengue cases and the monthly temperature for years 2005-2009 in Dhaka City Corporation, Dhaka, Bangladesh. Figure S3: Autocorrelation and partial autocorrelation function plots for monthly rainfall and the monthly humidity for years 2005-2009 in Dhaka City Corporation, Dhaka, Bangladesh.
