# Supplementary material for: An Association between Rainy Days with Clinical Dengue Fever in Dhaka, Bangladesh: Findings from a Hospital Based Study
Source: Int J Environ Res Public Health. 2020 Dec 18;17(24):9506. doi: 10.3390/ijerph17249506 (PMC7765799; doi:10.3390/ijerph17249506)
Supplement: Supplementary file 1 [file ijerph-17-09506-s001.pdf]

Supplementary Document

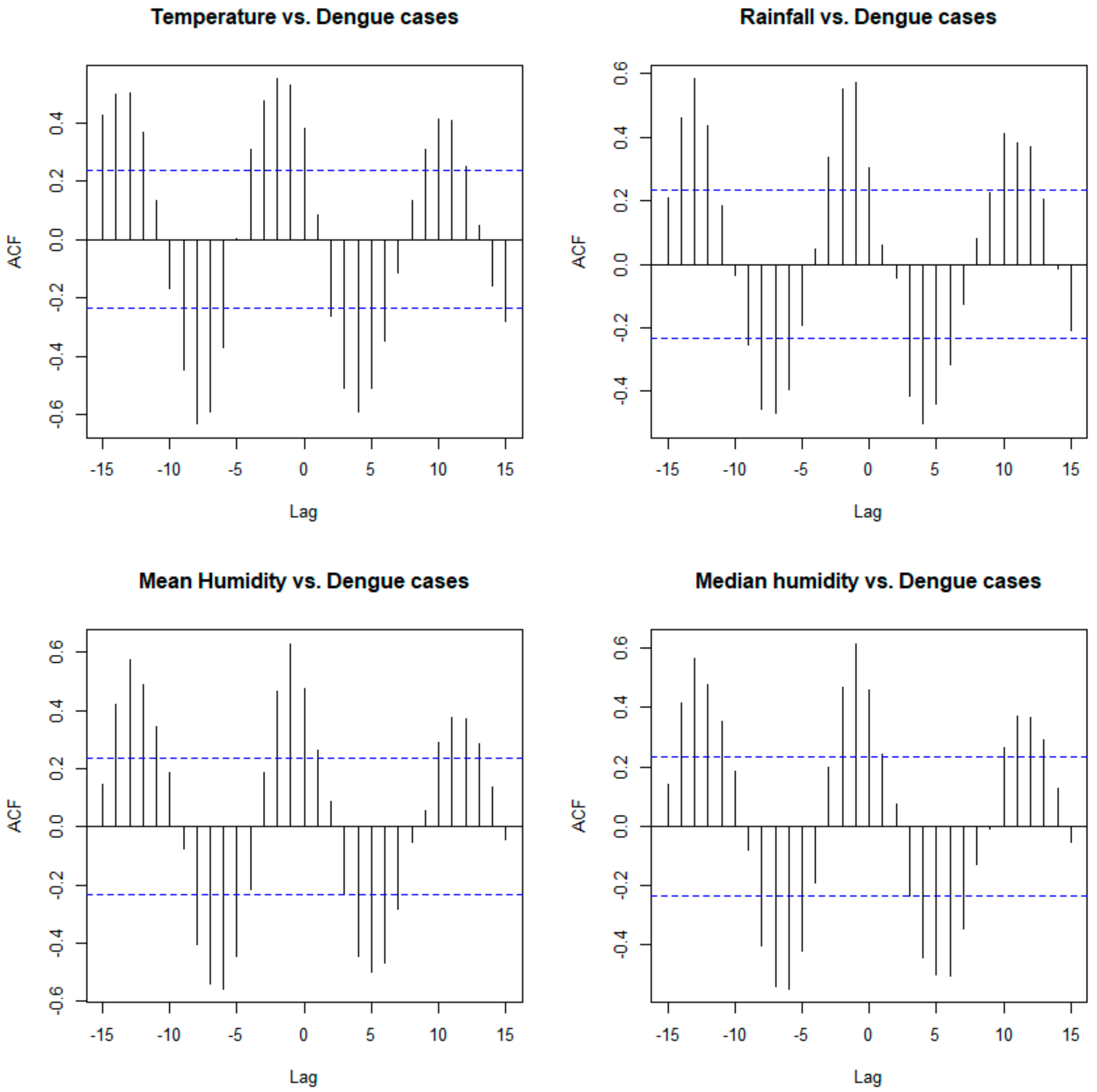

Figure S1. Cross Correlation Function (CCF) plot for different climate variables and the total number of monthly dengue cases for years 2005-2009 in Dhaka City Corporation, Dhaka, Bangladesh.

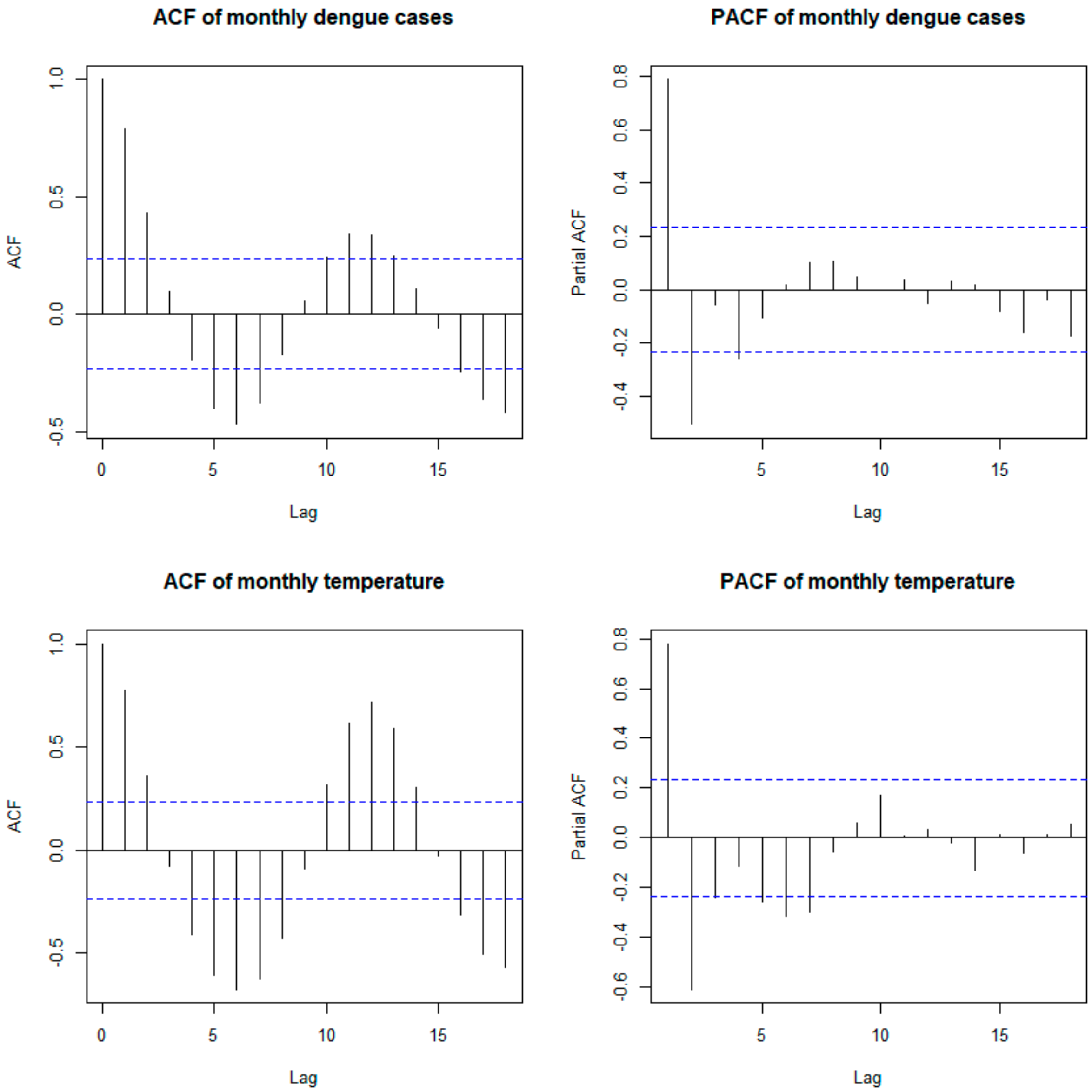

Figure S2. Autocorrelation and partial autocorrelation function plots for total number of monthly dengue cases and the monthly temperature for years 2005-2009 in Dhaka City Corporation, Dhaka, Bangladesh.

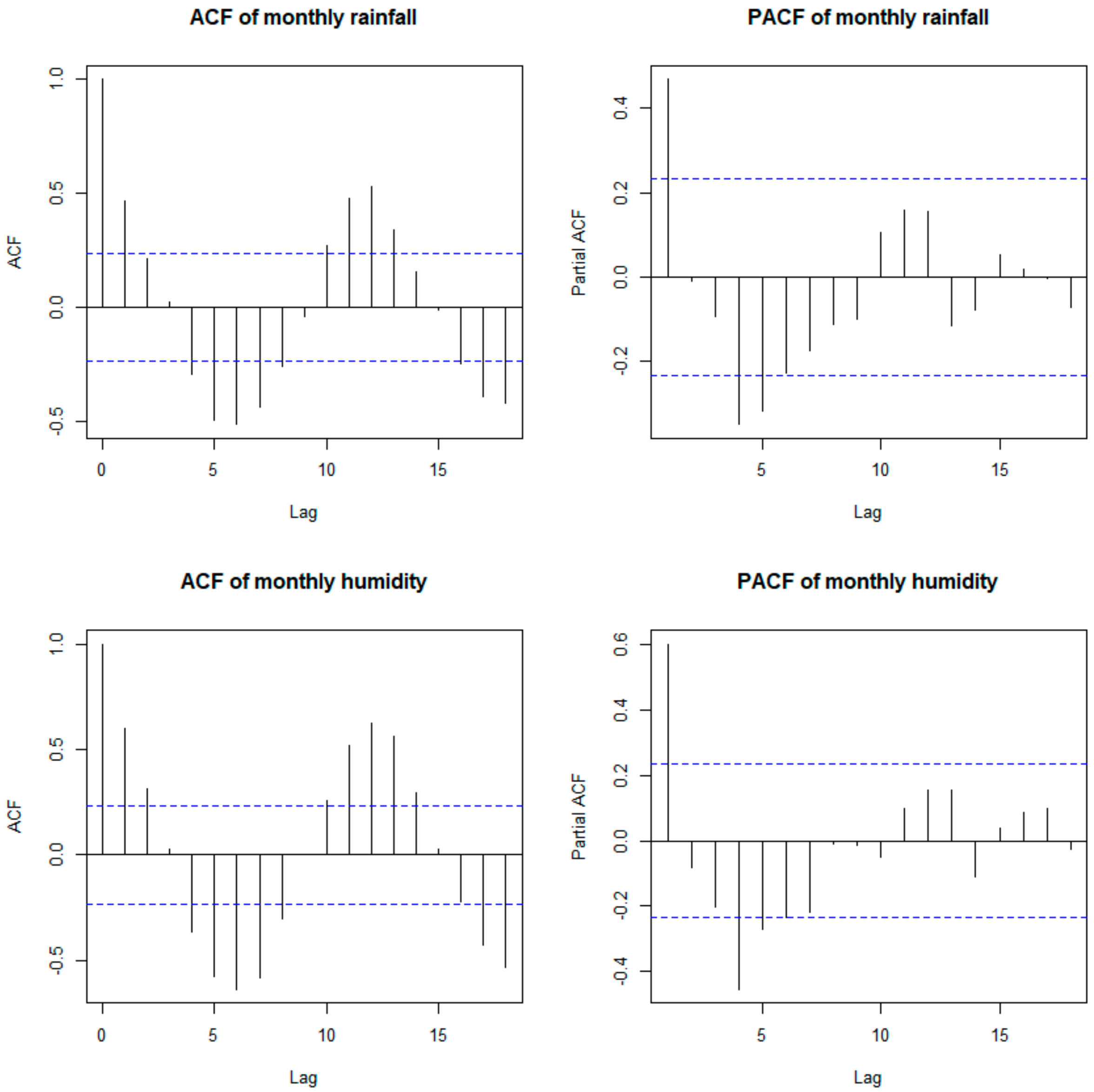

Figure S3. Autocorrelation and partial autocorrelation function plots for monthly rainfall and the monthly humidity for years 2005-2009 in Dhaka City Corporation, Dhaka, Bangladesh.
